# Supplementary material for: The relationships of spiritual health, pregnancy worries and stress and perceived social support with childbirth fear and experience: A path analysis
Source: PLoS One. 2023 Dec 7;18(12):e0294910. doi: 10.1371/journal.pone.0294910 (PMC10703247; doi:10.1371/journal.pone.0294910)
Supplement: Attachment — Submitted filename: answer.docx [file pone.0294910.s002.docx]

Dear Editor,

Thank you for your attention.

We have corrected all your comments and submitted them in the supporting file.

Regards
