# Supplementary material for: The relationships of spiritual health, pregnancy worries and stress and perceived social support with childbirth fear and experience: A path analysis
Source: PLoS One. 2023 Dec 7;18(12):e0294910. doi: 10.1371/journal.pone.0294910 (PMC10703247; doi:10.1371/journal.pone.0294910)
Supplement: Attachment — Submitted filename: reviewer.docx [file pone.0294910.s003.docx]

The relationship of spiritual health, pregnancy worries and stress, and perceived social support with childbirth fear and experience: A path analysis

1. What about related studies? In addition, what is gap of the previous studies in these issues?
2. Why researchers used general social support scale for pregnant women? The study needs specific scale in this issue.
3. Considering the questionnaire length in this study and in pregnant women? What about arrangements in the study to prevent the accuracy decrease?
4. Why researchers used online consent form?
5. "Assuming	the correlation between the social support and fear and experience of childbirth, at least 0.16"author express reference?
6. Persian Version of the Pregnancy Worries and Stress Questionnaire (PWSQ)
7. This tools generally was used in assessing anxiety ?
8. "The second stage was performed after the delivery. At this stage, one week before the probable date of the delivery, the mother was contacted via a phone call and asked to inform the researcher when they attended the hospital. The researcher then visited the hospital to collect information on the mother's childbirth experience and perceived social support when she went to the postpartum ward and was in a more stable condition. If a mother's clinical condition were not suitable for any reason, the completion of the questionnaires would be postponed until the mother's condition stabilized". If women did 9.Which, kind of social support (instrumental support-emotional – information- appraisal) had the highest negative causal relationship with fear in pregnant women?
