# Supplementary material for: The relationships of spiritual health, pregnancy worries and stress and perceived social support with childbirth fear and experience: A path analysis
Source: PLoS One. 2023 Dec 7;18(12):e0294910. doi: 10.1371/journal.pone.0294910 (PMC10703247; doi:10.1371/journal.pone.0294910)
Supplement: Attachment — Submitted filename: Respound to reviewer.docx [file pone.0294910.s005.docx]

Dear Editor

Thank you for your valuable comments. We corrected and answerd all of them as follows:

Reviewer #2: 1. There are some conflicting statements in the manuscript. For example, in Limitation section, authors said “Another limitation was the cross-sectional nature of the study that deprives the researcher from the opportunity to follow up ideas.” However, in Methods section, this longitudinal (prospective) study was performed in 2021 on eligible pregnant mothers presenting to the selected health centers in Qazvin, Iran. What is the kind of this study design actually? Besides, in Limitation section, part of the study was done after delivery, which may have tired mothers and affected their responses. But in Methods section, if a mother's clinical condition were not suitable for any reason, the completion of the questionnaires would be postponed until the mother's condition stabilized. Limitation section is very important for the current study and future research. Authors should take more efforts to improve it.

Answer: Thank you for your attention. This study was longitudinal and we corrected. We rewrite limitation part, and highlighted.

2. The Conclusion section can hardly cover main findings of this study. It is very simple and unspecific. For example, in Conclusion section, our study showed that spiritual health, and perceived social support is related to childbirth fear and experience in pregnant mothers. However, if authors want to get this finding, it is not necessary to conduct a path analysis. On the other hand, the third aim of this study is to explore what is the childbirth experience (direct/indirect) on fear of childbirth in pregnant mothers? The fifth aim is to explore if pregnancy worries and stress mediate the effect of fear of childbirth on childbirth experience? These research questions are not reflected well in the subsequent sections, such as Results section, Discussion section, and Conclusion section. In other words, these sections seem like they come from different researches and different authors. Authors should try to make different sections of the manuscript become closely related.

Answer: Thank you for your attention. If we only wanted to determine the relationship between this variables, yes it wasn’t need to use path analysis, but, we wanted to understand the direct effect and indirect effect of these variables, so we had to do the powerful analysis like path analysis.About third and fifth questions, we added them in result ,discussion and conclusion parts and highlighted.

Thanks

Reviewer #3: Dear Editor,

1. What is the total number of pregnant women followed in the center where the study was conducted?

## Answer: Thank you for your attention. In this study Two Referral Center of Qazvin were selected) Kowsar and shafa hospitals) and 352 eligible pregnant mothers were assessed. We added it in methods part and highlighted.

## 2. It would be appropriate to indicate the dates of the study and the duration of the study.

## Answer: Thank you for your comment. This study was conducted from 2021/3/3 to 2021/9/2 .we added in methods part and highlighted.

## 3. Were there any cases not included or excluded from the study? How many and why?

## Answer: Thank you for your comment. We didn’t have any case that not included or excluded from study.

## 4. Women who have fear of childbirth are often affected by their “delivery choice”. It is stated that the fear of childbirth increases the rate of cesarean section and that a large part of women prefer cesarean delivery instead of vaginal delivery. Was the mode of delivery examined in this study?

**Answer: Thank you for your comment.in this study we assessed all variable in path analysis. Path analysis requires the same type of data as linear multiple regression. In other words, you need a dependent variable that is continuous and normally distributed. ( Munro's statistical methods for health care research: Wolters Kluwer Health/Lippincott Williams & Wilkins; 2013) Accordingly, we couldn't assess the mode of delivery. Because of that, we only entered the mothers who wanted to have a vaginal delivery and did it.**

**5. Were the pregnancies of the pregnant women included in the study planned and desired pregnancies?**

**Answer: Thank you for your comment.** Yes, all pregnancies were planned and desired, and for this subject, first, we asked women about their pregnancy, and if it was wanted, they entered the study. We added it to the inclusion criteria**, and** highlighted.

**6. Another factor that causes fear of birth is previous negative birth and abortion experiences. Was it evaluated in this study?**

**Thank you for your comment. Yes, it was our inclusion criteria.**

**7. How the study was carried out is not fully understood. Were the cases included in the study evaluated twice, before and after birth? Were these interviews conducted face-to-face or over the phone? Were the scales and questionnaires given to the subjects and asked to be filled in at home? In this case, erroneous results may occur.**

**Answer: Thank you for your comment. Yes, as we wrote in the 2.3. Procedures: Data were collected in two stages in this study; the first stage was performed during the last four weeks of the pregnancy, and the second stage was performed after the delivery. The questionnaires were given to the participants to complete; if they could not complete the questionnaires in one meeting, the researcher would set the next time to complete the questionnaires, which means they came back to the center and completed, so all questionnaires were completed face-to-face and not at home.**

**8. It is recommended to expand the discussion section with the results of studies conducted in different cultures on the subject. The following studies can be used.
Chválna Zuzana, Dominová Natália, Ostatníková Michaela, et al. Prevalence and risk factors for serious birth concerns in unselected population of mothers. Prevalencia a rizikové faktory závažných obáv z pôrodu u neselektovanej populácií rodičiek. Ceska Gynekol. 2023;88(2):80-85. doi:10.48095/cccg202380
Nieminen K, Stephansson O, Ryding EL. Women's fear of childbirth and preference for cesarean section--a cross-sectional study at various stages of pregnancy in Sweden. Acta Obstet Gynecol Scand. 2009;88(7):807-813. doi:10.1080/00016340902998436

Viirman F, Hesselman S, Wikström AK, et al. Negative childbirth experience - what matters most? a register-based study of risk factors in three time periods during pregnancy. Sex Reprod Healthc. 2022;34:100779. doi:10.1016/j.srhc.2022.100779
Räisänen S, Lehto SM, Nielsen HS, Gissler M, Kramer MR, Heinonen S. Fear of childbirth in nulliparous and multiparous women: a population-based analysis of all singleton births in Finland in 1997-2010. BJOG. 2014;121(8):965-970. doi:10.1111/1471-0528.12599
Smith KB, Zdaniuk B, Ramachandran SO, Brotto LA. A longitudinal case-control analysis of pain symptoms, fear of childbirth, and psychological well-being during pregnancy and postpartum among individuals with vulvodynia. Midwifery. 2022;114:103467. doi:10.1016/j.midw.2022.103467**

**Answer: Thank you for your comment; we added and highlighted two references per your recommendation in the Discussion part.**

**9. It seems that the study was conducted during the period when the most important effects of COVID-19 were seen. The cases participating in the study may have been affected by this situation. It can be stated in the limitations of the study.**

**Answer: Thank you for your comment; we added it in the limitation part, and highlighted**

**10. Do the cases participating in the study have a previous history of psychiatric disorder and treatment?**

**Answer: Thank you for your comment. No. they didn’t have. This was our inclusion criteria as we wrote in inclusion criteria part:” not having a mental illness according to the self-reports or health records, and not taking antidepressants and anti-anxiety medications according to the self-reports or health records.”**

**11. Superiority and dissimilarity of this study from the other studies have not been emphasized enough. What is the difference should be emphasized**

**Answer: Thank you for your comment. As we wrote in the end of background:” To the best of the authors’ knowledge, perceived social support, spiritual and psychological variables, and fear and experience of childbirth have not been addressed yet in a single model.” It means this model can be first model that show casual paths between these variables.

12 .What is the recommendation for further studies?**

**Answer: Thank you for your comment. We wrote in the limitation part: “This study assessed pregnant mothers presenting to the selected health centers. Mothers who did not refer to the health center or offered in other specific centers did not set so that it could be another limitation. We recommended that in further research, they consider.” and also “The study was conducted during COVID-19 so the participating cases may have been affected by this situation” so ut can be done in another time.**

**Regards**

Researchers Team
