# Supplementary material for: The relationships of spiritual health, pregnancy worries and stress and perceived social support with childbirth fear and experience: A path analysis
Source: PLoS One. 2023 Dec 7;18(12):e0294910. doi: 10.1371/journal.pone.0294910 (PMC10703247; doi:10.1371/journal.pone.0294910)
Supplement: Attachment — Submitted filename: Response to Reviewers.docx [file pone.0294910.s006.docx]

Dear Editor

Thank you for your valuable comments. We corrected and answered all of them as follows:

1. We note that several of your files are duplicated on your submission. Please remove any unnecessary or old files from your revision, and make sure that only those relevant to the current version of the manuscript are included.

2. Please note that funding information should not appear in the Acknowledgments section or other areas of your manuscript. We will only publish funding information present in the Funding Statement section of the online submission form. Please remove any funding-related text from the manuscript.

Answer: Thank you for your comments. We corrected all.

Regards

Researchers Team
